# Plant Hormone-Mediated Regulation of Heat Tolerance in Response to Global Climate Change

**DOI:** 10.3389/fpls.2020.627969

**Published:** 2021-02-11

**Authors:** Ning Li, Dejuan Euring, Joon Yung Cha, Zeng Lin, Mengzhu Lu, Li-Jun Huang, Woe Yeon Kim

**Affiliations:** ^1^State Key Laboratory of Cultivation and Protection for Non-Wood Forest Trees, College of Forestry, Central South University of Forestry and Technology, Hunan, China; ^2^Forest Botany and Tree Physiology, University of Göttingen, Göttingen, Germany; ^3^Division of Applied Life Science (BK21PLUS), Plant Molecular Biology and Biotechnology Research Center, Gyeongsang National University, Jinju, South Korea; ^4^Laboratory of Forest Genetics and Plant Breeding, College of Forestry, Central South University of Forestry and Technology, Hunan, China; ^5^State Key Laboratory of Tree Genetics and Breeding, Key Laboratory of Tree Breeding and Cultivation of the State Forestry and Grassland Administration, Research Institute of Forestry, Chinese Academy of Forestry, Beijing, China; ^6^State Key Laboratory of Subtropical Silviculture, School of Forestry and Biotechnology, Zhejiang A&F University, Zhejiang, China

**Keywords:** heat stress, phytohormone, heat response, heat tolerance, signal transduction

## Abstract

Agriculture is largely dependent on climate and is highly vulnerable to climate change. The global mean surface temperatures are increasing due to global climate change. Temperature beyond the physiological optimum for growth induces heat stress in plants causing detrimental and irreversible damage to plant development, growth, as well as productivity. Plants have evolved adaptive mechanisms in response to heat stress. The classical plant hormones, such as auxin, abscisic acid (ABA), brassinosteroids (BRs), cytokinin (CK), salicylic acid (SA), jasmonate (JA), and ethylene (ET), integrate environmental stimuli and endogenous signals to regulate plant defensive response to various abiotic stresses, including heat. Exogenous applications of those hormones prior or parallel to heat stress render plants more thermotolerant. In this review, we summarized the recent progress and current understanding of the roles of those phytohormones in defending plants against heat stress and the underlying signal transduction pathways. We also discussed the implication of the basic knowledge of hormone-regulated plant heat responsive mechanism to develop heat-resilient plants as an effective and efficient way to cope with global warming.

## Introduction

The world population is growing at an alarming rate and is forecast to reach nearly 10 billion by the middle of this century. Global food security has become a serious concern over recent years. Increasing agricultural crop productivity is a sustainable approach to feeding the future world population.

Plant growth and geographic distribution are severely limited by various abiotic stresses, such as drought, salinity, cold, and heat (Zhu, 2016). In particular, extreme seasonal heat caused by global warming substantially disturbs normal crop growth and yield around the world, which further exacerbates food insecurity and malnutrition. It is estimated that a 1°C increase in seasonal temperature may directly cause 2.5–16% staple crop yield losses in tropical and subtropical regions (Battisti and Naylor, 2009). Heat stress has deleterious influences on plant growth and development. Biochemical and physiological consequences following heat stress include excess accumulation of reactive oxygen species (ROS) that induces oxidative stress, irreversible denaturation of proteins that leads to protein misfolding and aggregation, and alterations to the lipid membrane that result in injured membrane permeability and raft disruption (Goraya et al., 2017; Lippmann et al., 2019). In addition, the photosynthesis system is highly sensitive to heat stress (Allakhverdiev et al., 2008; Wang et al., 2017a; Hu et al., 2020). High temperature induces a variety of damage to photosynthesis, ranging from moderate stress that principally attenuates photosynthetic rate to permanent impairment that eliminates photosynthetic capacity.

As sessile organisms, plants immediately sense nearby dangers but cannot escape from harmful environments. Plants have evolved an arsenal of adaptive mechanisms to achieve tolerance in order to survive under heat stress. Plants change their metabolism to increase antioxidant capacity to maintain cellular redox balance and homeostasis upon sensing stress (Nadarajah, 2020). The expression and accumulation of heat-shock proteins (HSPs) are enhanced as chaperones to protect proteins against heat-induced irreversible damage (Jacob et al., 2017; Ul Haq et al., 2019). Accordingly, cellular signaling cascades and transcriptional activities are activated to coordinate physiological and biochemical processes by gene expression changes in response to elevated temperature (Qu et al., 2013).

Phytohormones are the endogenous signal molecules that play an important role in almost every aspect of plant development, growth, and defense processes (Verma et al., 2016; Kumar et al., 2019; Emenecker and Strader, 2020; Jang et al., 2020; Küpers et al., 2020). In recent years, studies have found that exogenous application of phytohormones significantly ameliorated heat-induced damage and improved plant heat tolerance, which indicates that phytohormones actively participate in plant response to heat stress. The phytohormone biosynthetic and signaling pathways have been thoroughly elucidated, mainly in the model plant *Arabidopsis thaliana*. Investigation of the underlying molecular processes of plant hormone-mediated heat response may provide opportunities to generate thermotolerant varieties and to grow agriculturally important crop cultivars in response to changing climate (Grover et al., 2013). In this review, we summarize and discuss recent progress on the versatile roles and the molecular mechanisms of phytohormones involved in plant heat tolerance and how agricultural translational research may transfer the emerging knowledge to ensure global food security.

## Roles of Phytohormones in Plant Response to Heat Stress

### The Stress Hormone Abscisic Acid Improves Plant Tolerance to Heat Stress

Abscisic acid (ABA) is a phytohormone crucial for plant growth and regulates plant stress responses. In general, ABA limits plant growth in order to coordinate plant adaptation to stressful conditions, e.g., salinity, drought, cold, and heat (Suzuki et al., 2016).

Air temperatures exceeding certain threshold levels cause excessive oxidative stress and membrane damage, which collectively reduce plant photosynthetic and transpiration efficiencies (Bita and Gerats, 2013; Hasanuzzaman et al., 2013). Heat shock elicits a rapid and transient increase in endogenous ABA levels (Larkindale et al., 2005). ABA confers heat tolerance by increasing ROS levels to enhance antioxidant capacity. ABA induces the expression of plant NADPH oxidases, known as respiratory burst oxidase homologs (RBOHs), to induce ROS. RBOHs are plasma membrane proteins. By structural and phylogenetic analysis, 10 *RBOH* genes (*AtRBOHA-AtRBOHJ*) were identified in the *Arabidopsis* genome (Suzuki et al., 2011; Kaya et al., 2019). Transcriptional analysis revealed that only the expression of AtRBOHD, the main NADPH oxidase in *Arabidopsis*, was up-regulated in leaves upon heat stress (Suzuki et al., 2011). The At*RBOHD* loss-of-function mutant displayed impaired heat stress tolerance as measured by seed germination and seedling survival capacities (Larkindale et al., 2005; Silva-Correia et al., 2014). Exogenous application of ABA increases hydrogen peroxide (H_2_O_2_) accumulation. H_2_O_2_ mediates ABA-induced thermotolerance by elevating ROS scavenging enzymes and antioxidant substances. In the ABA biosynthesis-deficient mutant plants that lack ABA production, heat-inducible H_2_O_2_ accumulation is abolished. Consequently, the ABA-deficient mutant plants show impaired heat tolerance, which can be reversed by exogenous addition of ABA (Larkindale and Knight, 2002). Similarly, treatment with ABA synthesis inhibitor impairs heat response by reducing ROS levels in plants (Larkindale et al., 2005). Both ABA biosynthetic and signaling pathways are involved in heat stress response. In addition to ABA synthetic mutants, plants with mutation in ABA signaling components fail to establish thermotolerance and display increased sensitivity to heat stress (Larkindale et al., 2005). However, the molecular mechanism by which ABA mediates heat-induced expression of antioxidant related genes to enhance heat tolerance in plants is largely unclear.

Abscisic acid may also serve as a thermo-priming hormone that enables plants to respond more rapidly and efficiently to heat stress. ABA improves drought acclimation in plants. Exogenous application of ABA confers *Arabidopsis* resistance more rapid and effective to drought-triggered dehydration stress by priming a transcriptional memory (Virlouvet et al., 2014). ABA mediates plant tolerance to a variety of abiotic stressors and is also required for priming across different stressors (Sah et al., 2016). A mild and transient drought treatment (drought priming) enhanced heat tolerance in tall fescue (*Festuca arundinacea* Schreb.) and *Arabidopsis* (Zhang et al., 2019). Indeed, both drought priming and pretreatment of ABA could improve heat tolerance in tall fescue. ABA is required for drought priming-induced heat tolerance (Zhang et al., 2019), and the priming effect is compromised in ABA-deficient *Arabidopsis* mutant plants or in ABA-synthesis inhibitor-treated tall fescue plants.

Interestingly, ABA may modulate levels of carbohydrates and energy status through accelerated transport and enhanced metabolism of sucrose to strengthen plant thermal tolerance (Rezaul et al., 2019; Santiago and Sharkey, 2019). The expression of genes involved in sucrose transport and metabolism, such as sucrose transporters, sucrose synthase, and invertase, is activated by ABA under heat stress. However, sucrose alone may contribute to plant thermal adaption by providing energy and/or acting as a regulatory signal (Wind et al., 2010). Induction of gene expression, protein production, and ROS scavenging during heat responses are high energy cost processes. Therefore, ABA and sucrose show synergistic effects on improving plant heat tolerance.

Heat-shock proteins function as molecular chaperones to defend plants against heat stress by maintaining protein in functional conformations. Upon heat stress, HSPs are rapidly induced through the transcriptional activity of heat stress transcription factors (HSFs). ABA also improves plants’ heat tolerance through the regulation of HSFs and HSPs. For instance, exogenous ABA application alleviates heat-induced detrimental effects and enhances heat tolerance of tall fescue (Wang et al., 2017b). ABA treatment increases the expression levels of tall fescue heat stress transcription factor A2c (FaHSFA2c). Notably, the tall fescue ABA-responsive element binding protein 3 (FaAREB3), a master regulator of the ABA-responsive pathway, directly binds to the *cis*-elements in the promoter of the *FaHSFA2c* gene.

**FIGURE 1:**
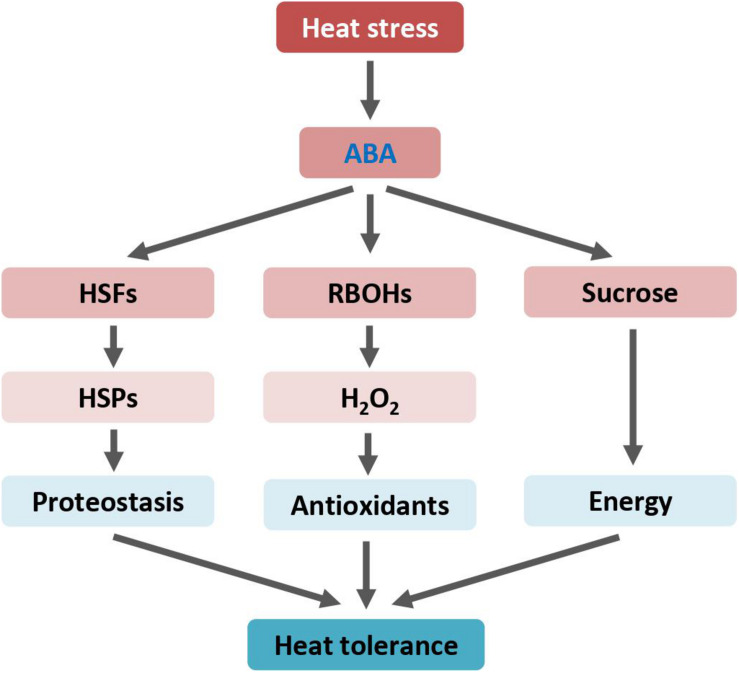
ABA enhances heat tolerance by mediating HSP and RBOH genes expression, and sucrose metabolism. Arrows indicate positive regulation. ABA, abscisic acid; H_2_O_2_, hydrogen peroxide; HSFs, heat shock transcription factors or heat shock factors; HSPs, heat shock proteins; RBOH, respiratory burst oxidase homologue.

Heat stress extensively up-regulates transcript levels of the wheat (*Triticum aestivum* L.) heat shock factor HsfA6f (TaHsfA6f) (Bi et al., 2020). *Arabidopsis* transgenic plants overexpressing the *TaHsfA6f* gene showed increased accumulation of ABA and subsequently improved tolerance to various environmental stresses, including heat. Further transcriptomic analysis revealed that, in addition to a number of heat-protective genes, several ABA biosynthesis and signaling genes are differentially expressed in *TaHsfA6f* transgenic plants when compared with non-transgenic plants. Under heat stressed conditions, ABA activates *TaHsfA6f* expression, and TaHsfA6f in return enhances ABA accumulation, forming a positive feedback circuit to strengthen heat response. Regulatory components of this circuit may serve as valuable targets for molecular breeding and genetic engineering to develop heat-resistant crops for securing future food production (Figure 1).

### The Growth Hormone Auxin Mediates Heat-Induced Plant Thermomorphogenesis

As an adaptive response to higher ambient temperatures, plants exhibit dramatic morphological and architectural changes termed thermomorphogenesis. The phytohormone auxin plays an important role in heat stress-induced thermomorphogenesis, including stem (hypocotyl) elongation and leaf hyponasty (Küpers et al., 2020). The heat-induced growth response is drastically restrained in auxin signaling mutants or transgenic plants expressing the bacterial *IAA-lysine synthase* (*iaaLys*) gene, which contains a relatively lower level of free IAA (Gray et al., 1998). Correspondingly, auxin concentration is significantly increased in seedlings grown under heat stress. However, exogenous auxin application does not trigger hypocotyl elongation at normal growth temperatures, suggesting that auxin accumulation is required but not sufficient for temperature-induced thermomorphogenesis (Gray et al., 1998).

The PIN-LIKES (PILS) proteins are putative auxin carriers at the endoplasmic reticulum (ER), where they are implicated in intracellular auxin distribution and limit nuclear auxin availability, and consequently confound auxin signaling output (Sauer and Kleine-Vehn, 2019). PILS6 is temperature-sensitive. Heat shock diminishes the PILS6 protein levels, resulting in subcellular auxin re-distribution and increase in auxin signaling response (Feraru et al., 2019). The Auxin Response Factors (ARFs) are involved in auxin-responsive hypocotyl elongation. ARFs activate auxin-responsive gene expression. The ARF-deficient plants displayed a decreased response to high temperatures (Reed et al., 2018).

HSP90 is required for plant thermomorphogenesis (Xu et al., 2012; di Donato and Geisler, 2019). Application of HSP90 inhibitor affects heat-induced hypocotyl elongation. HSP90 is required for the induction of auxin-responsive genes and the depletion of transcriptional repressors Aux/IAAs. In the auxin signaling pathway, Aux/IAAs interact with and restrain the transcriptional activity of ARFs. The HSP90 chaperone system stabilizes the auxin co-receptor F-box protein TIR1 at high temperatures (Wang et al., 2016; Watanabe et al., 2016).

**FIGURE 2:**
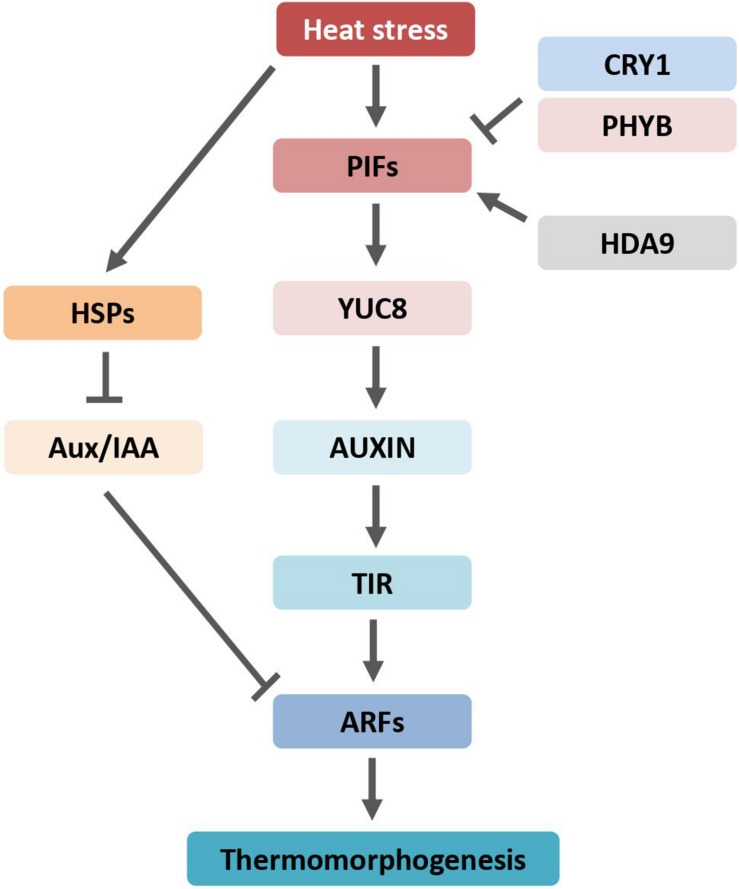
Auxin regulates plant morphogenesis through transcription factor PIFs and is counteracted by phyB and CRY1. Arrows indicate positive regulation, blunt-ended lines indicate negative regulation. ARF, AUXIN RESPONSE FACTOR; Aux/IAA, AUXIN/INDOLE-3-ACETIC ACID; HDA9, HISTONE DEACETYLASE 9; HSPs, heat shock proteins; phyB, phytochrome B; PIFs, PHYTOCHROME INTERACTING FACTORs; CRY1, cryptochrome 1; TIR1, TRANSPORT INHIBITOR RESPONSE 1; YUC8, YUCCA8.

Genetic studies showed that stem elongation and leaf hyponasty responses to heat stress require the activity of the basic helix-loop-helix (bHLH) transcriptional regulators Phytochrome Interacting Factor 4 (PIF4) and PIF7 (Koini et al., 2009; Fiorucci et al., 2020). The *Arabidopsis* PIF family contains eight members, namely, PIF1–8, which can interact with at least one of the phytochrome photoreceptors (Leivar and Monte, 2014; Pham et al., 2018). High-temperature-mediated thermomorphogenesis was abolished in *PIF4* and *PIF7* loss-of-function mutants. PIF4 and PIF7 activity depend on each other by forming heterodimers, whereas other PIFs play a neglectable, if any, role in *Arabidopsis* heat stress response. Auxin levels did not increase in *pif4* mutant plants at high temperatures (Franklin et al., 2011; Sun et al., 2012). The *pif4* mutants displayed dramatically reduced levels of auxin biosynthesis enzymes, such as members of YUCCA, aminotransferase, and cytochrome P450s, involved in temperature response. The expression of PIFs is also induced when plants are subjected to heat stress. An *in vitro* study showed that PIF4 directly binds to the promoter region of *YUCCA8* gene, a rate-limiting enzyme of auxin synthesis, and activates its expression (Sun et al., 2012). Therefore, PIFs play a major role in auxin-mediated thermomorphogenesis by controlling expression of auxin biosynthesis genes (Figure 2). In addition, PIFs also require components of the auxin signaling pathway to regulate high-temperature-induced hypocotyl growth. Interestingly, the chromatin-modifying enzyme Histone Deacetylation 9 (HDA9) is stabilized under high temperatures (van der Woude et al., 2019). HDA9 mediates histone deacetylation at *YUCCA8* nucleosomes to promote H2A.Z depletion and finally facilitates binding of a transcriptional regulator, such as PIF4, to the *YUCCA8* promoter (van der Woude et al., 2019).

Phytochrome interacting factor 4 is also involved in photomorphogenesis (Choi and Oh, 2016). The blue-light receptor cryptochrome 1 (CRY1) suppresses temperature-induced hypocotyl elongation through physical interaction with PIF4 and deterring the transcription activity of PIF4 (Ma et al., 2016). Heat induced auxin-responsive gene expression was abolished in *CRY1* ectopic expression plants. The potential thermosensor for thermomorphogenesis, phyB, phosphorylates PIF4, leading to the PIF4 protein degradation *via* the 26S proteasome pathway (Huq and Quail, 2002). Most recently, the epidermal auxin response was reported to be crucial for hypocotyl growth phenotype (Procko et al., 2016). Indeed, the endogenous PIF4 protein levels were increased particularly in epidermal cells by high temperatures (Kim et al., 2020). Ectopic expression of *PIF4* under the epidermis-specific promoter, but not under the vasculature-specific promoter, can restore the heat-induced hypocotyl growth in the *pifs* null mutants, indicating that epidermal PIF4 is required for thermomorphogenesis (Kim et al., 2020). Both auxin synthesis, perception, and signaling pathway are involved in heat-induced thermomorphogenesis in plants; thus, auxin enables a chance to generate climate-smart plants to ensure crop and food productivity in the context of global climate change.

### Brassinosteroid Regulates ROS Homeostasis and HSP Accumulation to Alleviate Heat Stress

Mazorra et al. (2011) examined how endogenous brassinosteroid (BR) content influences heat stress tolerance by assessing the ion leakage, lipid peroxidation, and survival rate after heat shock. The BR-deficient and -overproduction seedlings represented similar thermal tolerance, indicating that thermotolerance is independent of BR homeostasis, but downstream of BR signaling (Mazorra et al., 2011). In the spring barley (*Hordeum vulgare* L.), heat stress enhances the expression of HSPs. Compared to wild-type plants, proteins of the HSP group were less produced in the BR-deficient or BR-signaling mutants under heat stress (Sadura et al., 2020a).

Interestingly, the BR-induced HSP90 protein in turn interacts with two homologous transcription factors, BRI1-EMS-suppressor 1 (BES1) and brassinazole-resistant 1 (BZR1), of the BR signaling pathway (Shigeta et al., 2014, 2015; Samakovli et al., 2020). HSP90 may affect the stability of BES1 protein to facilitate BR-dependent gene expression (Samakovli et al., 2020). BR treatment leads to a significant increase in basic thermotolerance. Translation initiation and elongation factors of the translational machinery are present at significantly higher levels in BR-treated seedlings (Dhaubhadel et al., 2002). *In vivo* protein synthesis assay unraveled that increased accumulation of HSPs in BR-treated plants results from higher protein synthesis (Dhaubhadel et al., 2002). BR was also involved in regulating heat-induced accumulation of membrane proteins, such as proton-pumping ATPase and aquaporins (Sadura et al., 2020b). Heat stress triggers the translocation of *Arabidopsis* transcription factors bZIP17 and bZIP28 from the ER membrane into the nucleus, where they activate ER chaperone and BR signaling gene expression (Che et al., 2010).

Low levels of ROS may serve as second signals and thus play a regulatory role in plant stress response. Expression and activity of antioxidant enzymes are induced by exogenous BR treatment under heat stress (Nie et al., 2013). Like ABA, BR treatment in tomato (*Solanum lycopersicum* L.) leads to increases in *RBOH1* gene expression and H_2_O_2_ accumulation in leaf apoplast. Virus-induced gene silencing of *RBOH1* resulted in reduced H_2_O_2_ accumulation and compromised heat stress tolerance. Interestingly, H_2_O_2_ produced by RBOH1 activates MPK2, which in turn enhances *RBOH1* gene expression (Zhou et al., 2014). Therefore, BR-regulated heat stress tolerance includes a positive feedback loop among RBOH1, H_2_O_2_, and mitogen-activated protein kinase 2 (MPK2). However, the molecular mechanism by which BR induces *RBOH1* gene expression in not clear.

Brassinazole-resistant 1 is an important transcription factor of the BR signaling pathway (He et al., 2005). Following heat stress, BZR1 accumulates in the nucleus, where it regulates expression of growth-promoting genes (Ibañez et al., 2018). Yin et al. found that BZR1-like protein in tomato regulates heat response by directly controlling the receptor-like kinase FERONIA (FER) homologs (Yin et al., 2018). The promoter region of *FER2* and *FER3* contains several putative BZR1-binding sites. BZR1 binds to the promoters of *FER2* and *FER3* gene and activates their expression. The tomato *BZR1* loss-of-function mutant (*slbzr1*) was generated using CRISPR/Cas9 gene editing technology. Transcriptional analysis showed that *FER2* and *FER3* transcripts were induced by both BR and heat stress in the WT but not in the *slbzr1* mutant. Induction of RBOH1, production of apoplastic H_2_O_2_, and heat stress tolerance were impaired in the *FER2* and *FER3* gene-silenced plants (Yin et al., 2018). Consequently, BR-induced stress tolerance was diminished in those *FER2* and *FER3* gene-silenced plants.

Under heat stress, BZR1 was recruited to the promoter of *PIF4* gene and activated its expression (Ibañez et al., 2018). Furthermore, BZR1 was found to interact with the heat-activated transcription factor PIF4 in a transient bimolecular fluorescence complementation (BiFC) assay (Oh et al., 2012). Global chromatin immunoprecipitation sequencing (ChIP-Seq) analysis showed that BZR1 and PIF4 bind to common genomic targets. BZR1–PIF4 interaction regulates a core transcriptional network that integrates endogenous hormonal signals and environmental stimuli to modulate plant morphological development (Oh et al., 2012). The BR-receptor protein kinase BRI1 regulates root response to high temperatures (Martins et al., 2017). Elevated ambient temperatures specifically affect BRI1 levels at a post-transcriptional level to downregulate BR signaling and prompt root elongation.

### The Systemic Cytokinin Levels Positively Affect Heat Stress Tolerance

Numerous studies provide evidence that temperatures modulate cytokinin (CK) responses and CK levels are involved in plant adaptive mechanisms to temperature stress (O’Brien and Benková, 2013; Pavlů et al., 2018).

Hot ambient temperatures unusually cause pre-anthesis abortion in flower primordia of passion fruit (*Passiflora edulis*) during summers (Sobol et al., 2014). CK application showed an increased resistance in response to hot ambient temperatures. Genotypes isolated with higher CK in leaves can reach anthesis during summer. This result suggests that CK has a protective role for developing flowers exposed to heat stress and may have important implications in future crop breeding and field application to enhance crop production. CK applications can alleviate heat stress injury on creeping bentgrass (*Agrostis stolonifera* L.) (Wang et al., 2012). CK enhances antioxidant metabolism, by inducing activities of antioxidant enzymes superoxide dismutase, ascorbate peroxidase, and guaiacol peroxidase in roots under heat stress.

Heat stress treatment reduces panicle CK abundance and number of spikelets per panicle in rice. The heat stress severely decreases the xylem sap flow rate and CK transportation rate. Number of spikelets and CK content are positively correlated with CK translocation rates through xylem. CK applications alleviate the adverse impact of high temperatures on panicle differentiation and spikelet formation (Wu et al., 2017). Treatment of CK oxidase/dehydrogenase inhibitor showed a positive effect on heat stress tolerance in the model plant *Arabidopsis* (Prerostova et al., 2020). In addition, ectopic expression of the CK biosynthetic gene *isopentenyltransferase* (*ipt*) from the *Agrobacterium tumefaciens* increases CK levels, resulting in plant tolerance to heat stress (Skalák et al., 2016). A quantitative proteomic analysis was carried out to identify protein profiles in leaves and roots of *ipt* transgenic lines in response to heat stress. Expression of *ipt* resulted in protein changes involved in multiple functions, such as energy metabolism, protein compartmentation and storage, and stress defense. The identity of proteins altered in transgenic plants in response to heat stress provides further insights into the biochemical and molecular mechanisms of CK-regulated heat tolerance in plants (Xu et al., 2010).

A dramatic increase in CK levels and a transient decrease in ABA levels, therefore a higher CK/ABA ratio, were observed when shoots or whole plants were targeted to heat stress. The ABA levels in plants subjected to heat stress are under rigorous and dynamic control. Heat stress applied to part of plant elicits a rapid expression of components of CK signaling pathway in the non-exposed tissues. Heat-induced CK activates transcription of genes involved in photosynthesis and carbohydrate metabolism (Dobrá et al., 2015). Recently, an elegant proteomic study of *Arabidopsis* plants in response to high temperatures in the presence and absence of exogenous CK was performed to identify heat stress response proteins regulated by CK. A large proportion of the heat responsive proteome seems to be co-regulated by CK, indicating that heat stress and CK signaling pathway might be interconnected and CK directly involved in heat signaling in plants. Interestingly, the heat and CK response proteomes are preferentially targeted to the chloroplasts, which may play a major role in heat stress response (Cerný et al., 2014). Constitutive expression of a maize small *HSP* (*ZmsHSP*) in *Arabidopsis* under the control of CaMV 35S promoter causes lower endogenous CK content and higher sensitivity to CK during early developmental stage, indicating that *ZmsHSP* plays a role in CK response in plants (Cao et al., 2010).

Although climate change and global warming pose threats to forests, so far, research on the physiological and biochemical mechanisms that underlie heat stress response in woody trees remains scarce. An integrated physiological and phytohormonal profile of heat-induced thermotolerance in conifer, *Pinus radiata*, revealed that early heat shock and later heat tolerance exhibited differential dynamics patterns. CK plays important roles during long-term temperature acclimation and changes in plant developmental program to recover chloroplast function and photosynthetic ability (Escandón et al., 2016).

### SA Reduces Heat-Induced Growth and Physiological Damage

The role of SA in protecting plants against heat-induced damage was repeatedly reported. Exogenous SA treatment on alfalfa (*Medicago sativa* L.) seedlings notably alleviates heat shock-induced adverse effects. SA application prior to heat stress generally improved the plant growth and physiological activities, such as plant height, biomass, and photosynthetic efficiency (Wassie et al., 2020). Accordingly, SA reduces heat stress-induced membrane damage and modulates the activities of antioxidant enzymes including catalase (CAT), superoxide dismutase (SOD), and peroxidase (POD). Similarly, exogenous SA enhances tomato heat tolerance through improving photosynthesis efficiency and scavenging of reactive oxygen species by induction of antioxidants (Shah Jahan, 2019). However, SA has little, if any, influence on photosynthesis at normal growth temperatures. SA pretreatment alleviates the decrease of the net photosynthesis rate by protecting photosystem II function and maintaining higher Rubisco activities under heat stress (Wang et al., 2010). In addition, the chloroplast HSP21 proteins showed higher levels in both mock- and SA-treated leaves when stressed with heat shock. During the recovery period, the levels of HSP21 in SA-pretreated leaf samples remain high. SA not only relieves the decrease of photosynthesis rates under heat stress but also facilitates the recover of photosynthesis after stress, mainly due to maintaining higher levels of HSP21 chaperones in chloroplast.

**FIGURE 3:**
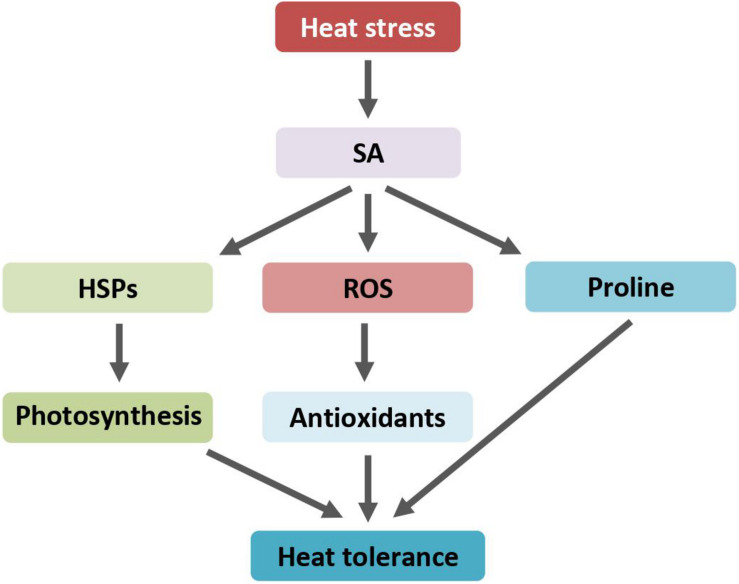
SA regulates heat response through induction of HSPs accumulation, ROS scavengers, and proline biosynthesis. Arrows indicate positive regulation. ROS, reactive oxygen species; SA, salicylic acid; HSPs, heat shock proteins.

Heat stress treatment induces the production of proline, which was further increased with exogenous SA application (Khan et al., 2013). The production of proline is an adaptive response that plants thrive for survival under adverse conditions (Verbruggen and Hermans, 2008; Szabados and Savouré, 2010). Proline acts as an antioxidant. SA significantly increases the activities of proline biosynthesis enzymes while inhibiting the activities of proline-metabolizing enzymes (Lv et al., 2011; Figure 3). Correlation between SA-induced protection of photosynthesis and SA-induced production of proline under heat stress implies that SA application ameliorates heat stress-induced oxidative stress apparently through maintaining a higher proline accumulation. On the contrary, using transgenic *Arabidopsis* plants overproducing proline by ectopically expressing a proline biosynthesis gene, it was found that increased proline production decreases plant thermotolerance under heat stress (Lv et al., 2011). The growth of transgenic *Arabidopsis* was more severely inhibited than that of control plants after heat shock. The inhibitory effect is attributed to the production of proline. The discrepancy between SA-induced endogenous proline accumulation and transgene-mediated proline overproduction on plants’ heat response is yet unclear.

Recently, it was reported that simultaneous application of both SA and melatonin mitigated the effects of heat stress by restoring relative water contents and increasing antioxidant enzyme activities in the aromatic herbs such as mint (*Mentha* × *piperita* and *Mentha arvensis* L.) that are cultivated worldwide, mainly in subtropical and tropical regions (Haydari et al., 2019). In the meantime, SA and melatonin treatment improves essential oil yields. The results could be considered for future applications in managing plants that are suffering from temperature extremes in these areas in the wake of global warming.

### Diverse Roles for JA and ET in Plant Heat Response

The gaseous hormone ethylene (ET) and the oxylipin-based hormone jasmonate (JA) together play multifaceted roles in plant response to biotic and abiotic stresses (Zhu, 2014). Both ET and JA are necessary for the activation of defense response against necrotrophic pathogens. The ET- and JA-mediated defense signaling pathways act synergistically to induce the expression of pathogen defense gene plant defensin 1.2 (PDF1.2) (Penninckx et al., 1998). Mutation in either ET- or JA-biosynthetic pathway renders plants hypersensitive to necrotrophic pathogens, such as *Botrytis cinerea* (Thomma et al., 1998, 1999). However, the ET and JA pathway may also act antagonistically to regulate plant adaptation to various abiotic stresses (Li et al., 2019).

**FIGURE 4:**
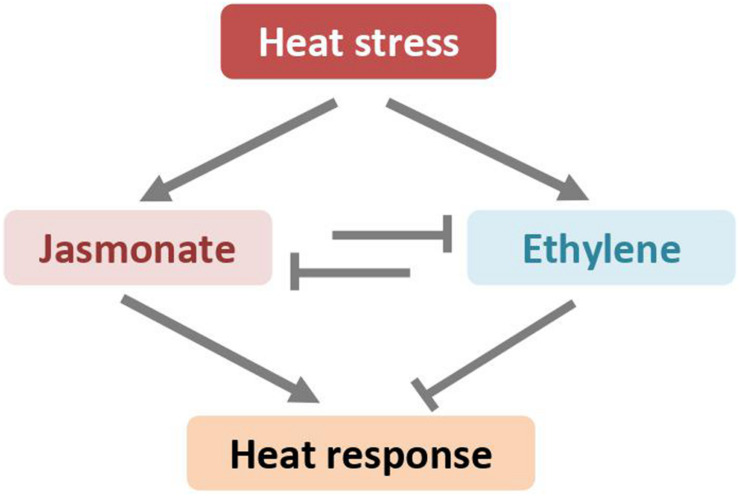
High-temperatures induced jasmonate and ethylene antagonistically regulates plant heat response through unknown mechanisms.

The abovementioned studies in the model plant *A. thaliana* also showed that high temperatures led to accumulation of both JA and ET (Larkindale and Knight, 2002; Larkindale et al., 2005). JA and ET show reverse effects on plant heat response (Figure 4). The *constitutive expresser of PR1* (*cpr5-1*) mutant, in which the signaling pathways of SA, JA, and ET are constitutively active, displays enhanced tolerance of heat stress (Clarke et al., 2009). However, the thermotolerance become compromised when *cpr5-1* crossed with mutants deficient in JA biosynthesis pathway (i.e., *jar1-1*) or in JA signaling pathway (i.e., *coi1-1*), demonstrating that at least JA is required for facilitating heat tolerance (Clarke et al., 2009). Indeed, the *coi1-1* mutant plants are thermosensitive and more susceptible to heat stress, as manifested by higher electrolyte leakage and severer chlorosis. Exogenous application of JA to wild-type plants before heat stress reduces heat-induced adverse damage, indicating that JA directly protects plants from heat stress (Clarke et al., 2009). However, the expression of HSPs, the well-established markers for thermotolerance, is neither induced by exogenous JA nor impaired in the JA signaling mutant. Although the role of JA in plant heat tolerance is well documented, the underlying mechanisms are not well understood (Sharma and Laxmi, 2015). Several lines of evidence suggest that JA might regulate plant heat response through a subset of JA-inducible transcription factors of the WRKY superfamily (Li et al., 2010, 2011; Dang et al., 2013).

In addition, JA represses stomatal development and induces stomatal closure, which is not suitable to sustain leaf cooling *via* transpiration at high ambient temperatures. It is also interesting to evaluate how heat stress may influence JA signaling pathway. Notably, JA has been reported to play a vital role in *Arabidopsis* cold response by regulating the C-repeat binding factor (CBF) pathway (Hu et al., 2013, 2017).

In contrast to JA, the ET appears to be a negative regulator on plant heat stress response in the model plant *Arabidopsis*. The *Arabidopsis* ET-insensitive mutant, *ethylene-insensitive 2-1* (*ein2-1*), which lacks a central regulator gene of ethylene signaling pathway, exhibits enhanced tolerance in response to heating (Clarke et al., 2009). Although heat stress elicits ethylene production, the ethylene-initiated and EIN2-mediated signaling pathway might repress plant heat response. Recently, Pan et al. provided evidence that ET biosynthesis and signaling are required for CO_2_-induced heat stress response in tomato (Pan et al., 2019). The airborne ET may reduce thermotolerance of holm oak (*Quercus ilex*) plants to heat stress by deterring antioxidant defenses (Munné-Bosch et al., 2004).

High temperatures during the reproductive stage cause severe threats to crop seed production. In pea (*Pisum sativum* L.), ethylene biosynthesis is differentially regulated in floral and fruit tissues upon heat stress in order to optimize resource allocation in reproductive tissues (Savada et al., 2017). In rice (*Oryza sativa* L.), ethylene confers thermotolerance and ameliorates heat-induced adverse effects (Wu and Yang, 2019). Therefore, the physiological, biochemical, and molecular functions of ET in plant in response to heat stress varied in plant species and tissues. A fuller understanding of the role of ET in plant thermotolerance must await further studies.

## Concluding Remarks and Future Perspectives

**FIGURE 5:**
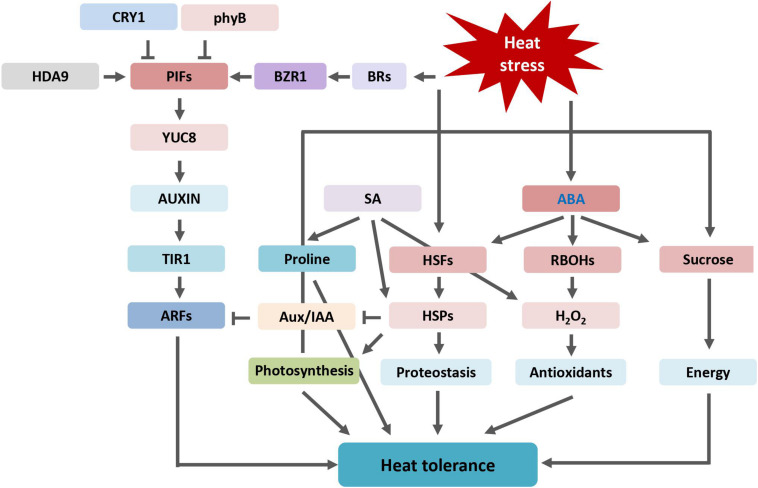
Overview of phytohormones regulated heat stress tolerance described and discussed in this review. ABA, abscisic acid; ARF, AUXIN RESPONSE FACTOR; Aux/IAA, AUXIN/INDOLE-3-ACETIC ACID; CRY1, BRs, brassinosteroids; BZR1, BRASSINAZOLE-RESISTANT 1; cryptochrome 1; HDA9, HISTONE DEACETYLASE 9; H_2_O_2_, hydrogen peroxide; HSFs, heat shock transcription factors or heat shock factors; HSPs, heat shock proteins; phyB, phytochrome B; PIFs, PHYTOCHROME INTERACTING FACTORs; RBOHs, respiratory burst oxidase homologues; SA, salicylic acid; TIR1, TRANSPORT INHIBITOR RESPONSE 1; YUC8, YUCCA8.

The implication of phytohormones in plant heat tolerance has been well-documented. An overview of phytohormones and pathway components involved in plant heat stress tolerance is shown in Figure 5. High temperatures stimulate the biosynthetic pathways resulting in higher accumulation of those hormones. Auxin and auxin pathway regulate plant thermomorphogenesis in response to heat stress to coordinate plant growth and stress defense. Both ABA and SA alleviate the negative effects of heat stress on plants by reducing oxidative damage and maintaining photosynthesis. In addition to SA, JA contributes to thermotolerance in *Arabidopsis* by physiological protection from heat-induced damage. CK alters antioxidant metabolism by inducing activities of antioxidant enzymes to alleviate heat stress injury. The role of ET on plant heat response is complicated and varies in different plant species. BR enhances plant thermotolerance by increasing photosynthetic rate and elevating the expression level of HSPs. BR signaling pathway induces the expression of PIFs and coordinates plant architectural changes under thermal stress condition. Heat stress inevitably causes accumulation of ROS. Thus, most hormones modulate plant ROS homeostasis, by production of antioxidants and scavenging of ROS, to improve heat tolerance.

Overwhelming evidences support the fact that plant hormones play important roles in plant biochemical, physiological, and architectural responses to high temperatures. The molecular mechanisms by which phytohormones regulate those defensive response are hitherto poorly understood. The signal transduction pathway leading to activation of hormone biosynthesis at high temperatures remains elusive. Crop plants are always exposed to a complex of environmental stresses in the field. Moreover, those hormones do not work along or act in a linear pathway to regulate plant growth, development, and defense. Intensive crosstalk between SA and ET/JA signaling pathways has been revealed in plant defensive response to pathogenic stress. The interaction and communication between multiple hormones in order to precisely coordinate plant defense response to heat stress deserve further investigation. For instance, it is unclear how JA and SA overcome ET-rendered negative effect to enhance heat tolerance in the *cpr5* mutant plants.

In the past decades, the heat-related responses in plants have been intensively studied (Bokszczanin et al., 2013; Hasanuzzaman et al., 2013; Ohama et al., 2017). So far, the molecular breeding and genetic modification strategies of developing heat-resilient agricultural crops are most unsuccessful, in a larger part due to limited knowledge on the molecular mechanism underlying plant heat response. Considering the elevated environmental temperature following global climate change that threatens plant growth, crop yield, and food productivity worldwide, there is a pressing need to thoroughly investigate the thermal-responsive hormone signal transduction pathway and sophisticated crosstalk between different signaling pathways to elucidate phytohormone function in plant heat response.

## Author Contributions

NL, L-JH, and WK: conceptualization. NL and ZL: literature review. NL, DE, and L-JH: writing—original preparation. ML and WK: writing—review and editing. NL, DE, JC, and ZL: design and revision of the images. All authors have read and agreed to the final version of the manuscript.

## Conflict of Interest

The authors declare that the research was conducted in the absence of any commercial or financial relationships that could be construed as a potential conflict of interest.
